# Complementing the US Food and Drug Administration Adverse Event Reporting System With Adverse Drug Reaction Reporting From Social Media: Comparative Analysis

**DOI:** 10.2196/19266

**Published:** 2020-09-30

**Authors:** Zeyun Zhou, Kyle Emerson Hultgren

**Affiliations:** 1 College of Pharmacy Purdue University West Lafayette, IN United States

**Keywords:** adverse drug reactions, FAERS, social media reporting, pharmacovigilance

## Abstract

**Background:**

Adverse drug reactions (ADRs) can occur any time someone uses a medication. ADRs are systematically tracked and cataloged, with varying degrees of success, in order to better understand their etiology and develop methods of prevention. The US Food and Drug Administration (FDA) has developed the FDA Adverse Event Reporting System (FAERS) for this purpose. FAERS collects information from myriad sources, but the primary reporters have traditionally been medical professionals and pharmacovigilance data from manufacturers. Recent studies suggest that information shared publicly on social media platforms related to medication use could be of benefit in complementing FAERS data in order to have a richer picture of how medications are actually being used and the experiences people are having across large populations.

**Objective:**

The aim of this study is to validate the accuracy and precision of social media methodology and conduct evaluations of Twitter ADR reporting for commonly used pharmaceutical agents.

**Methods:**

ADR data from the 10 most prescribed medications according to pharmacy claims data were collected from both FAERS and Twitter. In order to obtain data from FAERS, the SafeRx database, a curated collection of FAERS data, was used to collect data from March 1, 2016, to March 31, 2017. Twitter data were manually scraped during the same time period to extract similar data using an algorithm designed to minimize noise and false signals in social media data.

**Results:**

A total of 40,539 FAERS ADR reports were obtained via SafeRx and more than 40,000 tweets containing the drug names were obtained from Twitter’s Advanced Search engine. While the FAERS data were specific to ADRs, the Twitter data were more limited. Only hydrocodone/acetaminophen, prednisone, amoxicillin, gabapentin, and metformin had a sufficient volume of ADR content for review and comparison. For metformin, diarrhea was the side effect that resulted in no difference between the two platforms (*P*=.30). For hydrocodone/acetaminophen, ineffectiveness as an ADR that resulted in no difference (*P*=.60). For gabapentin, there were no differences in terms of the ADRs ineffectiveness and fatigue (*P*=.15 and *P=*.67, respectively). For amoxicillin, hypersensitivity, nausea, and rash shared similar profiles between platforms (*P*=.35, *P=*.05, and *P=*.31, respectively).

**Conclusions:**

FAERS and Twitter shared similarities in types of data reported and a few unique items to each data set as well. The use of Twitter as an ADR pharmacovigilance platform should continue to be studied as a unique and complementary source of information rather than a validation tool of existing ADR databases.

## Introduction

### Background

Adverse drug reactions (ADRs) are the unintended effect of medicine at doses used for prophylaxis, diagnosis, or treatment [1]. ADRs can occur anytime when a patient takes a medication. Factors including drug and food interactions, medication errors, allergies, and metabolism contribute to the occurrence of ADRs. ADRs have been identified as one of the leading causes of death in the United States. ADRs resulted in more deaths than the pulmonary diseases, diabetes, HIV/AIDS, and pneumonia [2,3]. A systematic review on ADR-induced hospital admissions found that 5.3% of hospital admissions were associated with ADRs [4]. New drug therapies, the aging population, and polypharmacy expose the population to increased risks of ADRs [5]. The burden of ADRs necessitates appropriate detection and assessment, and reporting is fundamental to successful pharmacovigilance systems.

The US Food and Drug Administration (FDA) Adverse Event Reporting System (FAERS) is a database for reports of adverse events, medication errors, and product quality complaints [6]. Although FAERS serves as a valuable data source for postmarket pharmacovigilance, only drug manufacturers are required to send reports received from health care professionals and consumers to the FDA. Health care professionals and consumers may voluntarily submit reports, which may lead to incomplete data in FAERS. In order to obtain more comprehensive information on drug products, multiple data sources should be used to fill the information gap.

Social media has been proposed as a potential data source as it allows an easily accessible information sharing platform with almost no chronological and geographical constraints. A systematic review of 51 studies compared ADR reports on social media and other pharmacovigilance systems, and the review noted that the prevalence of all ADR reports ranged from 0.2% to 8% and social media contained more reports of mild ADRs than severe ADRs [7]. Previous studies showed that ADRs were underrepresented in clinical trial data, and less severe ADRs were more frequently reported on social media. Social media ADR reports reflected the ADRs reported on FAERS on average 11 months earlier [8,9]. Comparative studies suggested the practicality of using social media as a complementary resource and demonstrated a moderate agreement on ADR data between social media and FAERS [10,11]. These studies have shed light on the role of social media in ADR reporting. However, many studies only examined one or two less commonly used pharmaceutical agents, and some included more than 1000 drugs. While the inclusion tested a general scheme of social media reporting, it overlooked the role of social media reporting for common drugs.

The Center for Medication Safety Advancement (CMSA) at Purdue University College of Pharmacy aims to adopt previous research strategies and compare ADR reports in social media and FAERS. Twitter was selected as the social media for evaluation thanks to its simplicity and timeliness in information sharing and access. Twitter users can report an ADR in one tweet pursuant to the FDA guideline, which requires as a minimum dataset to constitute a viable report an identifiable patient, an identifiable reporter, a product exposure, and an adverse event [12]. Additionally, the FDA does not require reports to demonstrate causation or to be specific regarding the type of error. All suspected medication errors, ADRs, or adverse events are accepted as reports. Given the advantage of the Twitter database, the objective of this study is to validate the accuracy and precision of the research methodology and conduct evaluations of social media ADR reporting via tweets for commonly used pharmaceutical agents.

### Ethics Statement

All social media data used in data collection and analysis were extracted from public sources. Example tweets were paraphrased and edited to prevent unmasking through a reverse search on Twitter. FAERS reports on SafeRx were also anonymized. As data used in this study were publicly available, no institutional review board approval was sought.

## Methods

### Overview

**Figure 1:**
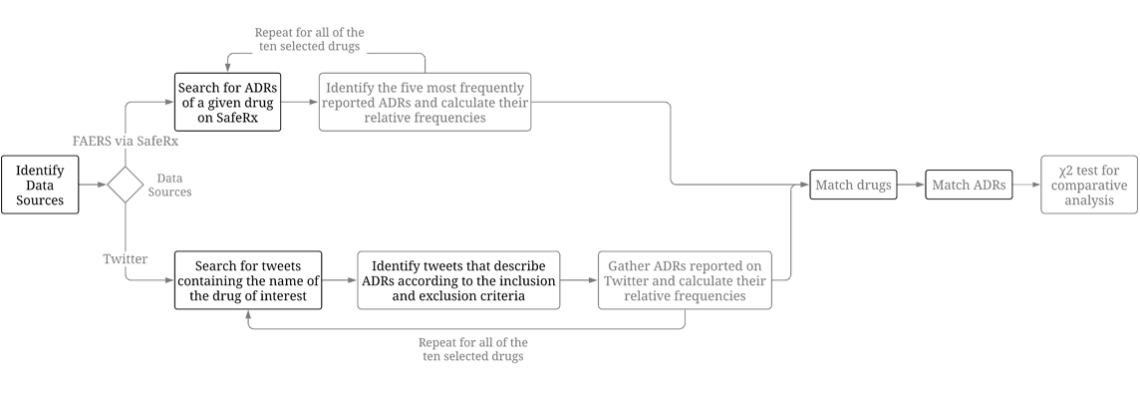
Methodology scheme. ADR: adverse drug reaction; FAERS: FDA Adverse Event Reporting System.

This study was divided into 3 sections: drug selection, FAERS data collection, and Twitter data collection. Collecting FAERS data included searching for ADR reports of a pharmaceutical agent and calculating relative frequencies of the 5 most frequently reported ADRs, whereas Twitter data collection required an additional step to identify relevant tweets according to inclusion and exclusion criteria. Figure 1 demonstrates the overall scheme for the methodology of this study.

### Pharmaceutical Agents

To identify the 10 most popular prescribed medications, prescription data were used from GoodRx, a health care company that operates a telemedicine platform. GoodRx generates a list of the top 10 drugs from monthly claims submitted by pharmacies in the United States; in November 2017, those drugs were hydrocodone/acetaminophen, levothyroxine, prednisone, lisinopril, amoxicillin, gabapentin, metformin, atorvastatin, alprazolam, and amlodipine [13]. Previous studies included both brand and generic names in data collection to expand the data that could be obtained [10,14]. Some studies further suggested that patients tended to use the most common brand name in social media if a drug had multiple brand names [15,16]. Accordingly, this study included common brand names in the searching criteria as Twitter users could be discussing ADRs using common brand names. For the data collection purpose of this study, the most commonly used brand name for each selected drug was identified according to Micromedex: Norco for hydrocodone/acetaminophen, Synthroid for levothyroxine, Deltasone for prednisone, Prinivil for lisinopril, Amoxil for amoxicillin, Neurontin for gabapentin, Glucophage for metformin, Lipitor for atorvastatin, Xanax for alprazolam, and Norvasc for amlodipine.

### US Food and Drug Administration Adverse Event Reporting System Data

Purdue University College of Pharmacy’s CMSA designed and maintained a searchable database for all published FAERS reports since 2012 under SafeRx. SafeRx enables large-scale studies to improve prescription medication safety as the database contains a collection of 4,935,048 ADRs, representing 294,652 different drugs from the fourth quarter of 2012 through December 2016. ADR reports were obtained via the FAERS Data Explore function in SafeRx. The search criteria were set to display data from March 1, 2016, to March 31, 2017, and the data included both brand and generic names of selected drugs as the primary suspect and the secondary suspect drug. After obtaining all ADR reports from SafeRx, the 5 most reported ADRs for each selected drug were recorded for data analysis.

### Twitter Data

Searchability and generalizability were the main factors in selecting Twitter as the social media platform. Twitter’s search engine enabled keyword-based searching within a predetermined time frame, and all public tweets containing the keyword could be displayed. According to the Pew Research Center, Twitter users were diverse in terms of age distribution and well balanced in terms of gender and geographic areas at the time of study in 2016 [17]. As medications including hydrocodone/acetaminophen, prednisone, levothyroxine could be prescribed to individuals from all age groups regardless of gender and geographic areas, Twitter’s population represented a robust data source for generalizability.

Tweets were obtained from the Advanced Search webpage on Twitter’s website [18]. Both generic and brand names of the selected medication were entered as keywords into the “any of these words” field in the Advanced Search engine. To exclude tweets containing advertisements, hyperlinks to external webpages, and retweets, characters including “rt” for retweets, “http,” and “.com” were entered into the “none of these words” field. By eliminating tweets from pharmaceutical companies, health care marketers, and agencies, Twitter data became more comparative to the FAERS data. Table 1 describes additional exclusion criteria in the selection of tweets. The “written in” field was set so that only tweets in the English language would be displayed. The time frame was chosen to be from March 2016 to March 2017 in order to correspond with the FAERS data obtained from CMSA’s SafeRx database. All tweets displayed were subsequently reviewed to include only those that described ADRs after consuming the medication. Those tweets served as the final source for data recording, which included the username, offending medication, content of the tweet, and types of ADRs. At the time of data collection, the number of tweets was benchmarked at 100 for analysis.

**Table 1:** Additional exclusion criteria in the collection of tweets.

Exclusion criteria	Examples
ADRs^a^ described a metaphorical narration instead of a true patient experience.	“He slept for a whole night like he took 20 Xanax”
ADRs occurred long before the date of tweeting.	“Lipitor gave me muscle aches when I took it 10 years ago”
Tweet was a part of copied lyrics, lines from books, and other forms of literature.	“Xanax got me sleeper. Leanin’ by the liter”
Tweet did not include the 4 minimal requirements to construct a report.	Tweets lacking the person who was reporting, the person who experienced the ADR, name of the drug, and the actual ADR.

^a^ADR: adverse drug reaction.

### Statistical Analysis

The analysis of ADR data from SafeRx and Twitter included the following components: calculation of relative frequencies, examination of ADR distribution, and test for association and independence. A chi-square test was used to statistically quantify the difference in ADRs between the FAERS data and Twitter data. It was appropriate to use the chi-square test as no cell in the cross-tabulation contained an expected value of 5 or below. The sample size required to achieve an a priori α<.01 was 96, and samples from both sources exceeded the threshold. The null hypothesis (H0) was “there is no significant difference between FAERS data and Twitter data on common ADRs.” The failure to reject H0 would signify that Twitter data were similar to and independent from the FAERS data. The statistical analysis in this study was conducted using SAS version 9.4 (SAS Institute Inc).

## Results

### US Food and Drug Administration Adverse Event Reporting System Data Result

A total of 40,539 FAERS ADR reports from March 1, 2016, to March 31, 2017, were obtained via SafeRx. Table 2 summarizes the 5 most reported ADRs for each of the 10 drugs.

**Table 2:** Five most frequently reported FDA Adverse Event Reporting System adverse drug reactions from March 1, 2016, to March 31, 2017, for each selected drug on SafeRx.

Drug and the top 5 adverse drug reactions		n (%)
**Hydrocodone/acetaminophen (Norco, n=1765)**		
	Ineffectiveness	429 (24.31)
	Nausea	371 (21.02)
	Fatigue	353 (20.00)
	Pain	345 (19.55)
	Headache	267 (15.13)
**Levothyroxine (Synthroid, n=3728)**		
	Fatigue	881 (23.63)
	Ineffectiveness	828 (22.21)
	Nausea	733 (19.66)
	Headache	664 (17.81)
	Diarrhea	622 (16.68)
**Prednisone (Deltasone, n=5689)**		
	Ineffectiveness	1423 (25.01)
	Fatigue	1332 (23.41)
	Dyspnea	1067 (18.76)
	Nausea	976 (17.16)
	Diarrhea	900 (15.82)
**Lisinopril (Prinivil, n=5386)**		
	Ineffectiveness	1243 (23.08)
	Fatigue	1172 (21.76)
	Diarrhea	1136 (21.09)
	Nausea	1062 (19.72)
	Dyspnea	773 (14.35)
**Amoxicillin (Amoxil, n=797)**		
	Hypersensitivity	328 (41.15)
	Fatigue	126 (15.81)
	Diarrhea	123 (15.43)
	Nausea	121 (15.18)
	Rash	99 (12.42)
**Gabapentin (Neurontin, n=5734)**		
	Ineffectiveness	1637 (28.55)
	Fatigue	1220 (21.28)
	Nausea	997 (17.40)
	Pain	966 (16.85)
	Diarrhea	914 (15.94)
**Metformin (Glucophage, n=5109)**		
	Hyperglycemia	1311 (25.66)
	Nausea	1111 (21.75)
	Ineffectiveness	973 (19.04)
	Diarrhea	919 (18.00)
	Fatigue	795 (15.56)
**Atorvastatin (Lipitor, n=6588)**		
	Type 2 diabetes	4601 (69.84)
	Hypersensitivity	586 (8.89)
	Fatigue	537 (8.15)
	Ineffectiveness	445 (6.75)
	Nausea	419 (6.36)
**Alprazolam (Xanax, n=2551)**		
	Ineffectiveness	561 (21.99)
	Fatigue	548 (21.48)
	Nausea	547 (21.44)
	Anxiety	451 (17.68)
	Headache	444 (17.40)
**Amlodipine (Norvasc, n=3192)**		
	Diarrhea	696 (21.80)
	Fatigue	682 (21.37)
	Ineffectiveness	636 (19.92)
	Nausea	611 (19.14)
	Dyspnea	567 (17.76)

### Twitter Data Result

More than 40,000 tweets containing the drug names as keywords from March 1, 2016, to March 31, 2017, were obtained from Twitter’s Advanced Search engine. Although searching on Twitter yielded an overall large quantity of tweets, ADRs of some drugs were simply not mentioned in enough tweets. Within the study period, searching keywords levothyroxine and Synthroid yielded 50 relevant tweets, keywords alprazolam and Xanax resulted in 35 relevant tweets, lisinopril and Prinivil were found in 33 relevant tweets, and only 3 relevant tweets were found for atorvastatin and Lipitor. No relevant tweets were found for keywords amlodipine and Norvasc. Due to the insufficiency of relevant tweets to meet the benchmark, the final Twitter data analysis did not include levothyroxine, alprazolam, lisinopril, atorvastatin, and amlodipine. Table 3 presents the ADRs reported for the remaining 5 drugs.

**Table 3:** Reported adverse drug reactions on Twitter from March 1, 2016, to March 31, 2017, for 5 drugs.

Drugs and adverse drug reactions		Value %
**Hydrocodone/acetaminophen**		
	Fatigue	36
	Ineffectiveness	22
	Pruritus	10
	Nausea	9
	Mood changes	5
	Vivid dreams	3
	Insomnia	3
	Headache	2
	Constipation	2
	Dizziness	2
	Chest tightness	1
	Delusion	1
	Hallucination	1
	Singultus	1
	Inattention	1
	Short-term amnesia	1
	Sweating	1
	Vomiting	1
**Prednisone**		
	Insomnia	25
	Increased appetite	23
	Mood changes	10
	Moon face	8
	Weight gain	8
	Fatigue	5
	Muscle weakness	4
	Jitteriness	3
	Diaphoresis	2
	Tachycardia	2
	Anxiety	2
	Bradycardia	1
	Cataracts	1
	Xerostomia	1
	Dyspnea	1
	Heartburn	1
	Osteoporosis	1
	Stomachache	1
	Visual hallucination	1
	Thirst	1
**Amoxicillin**		
	Hypersensitivity	46
	Rash	16
	Ineffectiveness	15
	Nausea	8
	Diarrhea	5
	Fatigue	3
	Pruritus	3
	Vomiting	3
	Stomachache	1
**Gabapentin**		
	Drowsiness	31
	Fatigue	24
	Ineffectiveness	23
	Weight gain	8
	Dizziness	5
	Nausea	2
	Blurred vision	1
	Dysphasia	1
	Confusion	1
	Headache	1
	Jitteriness	1
	Mood changes	1
	Vivid dreams	1
**Metformin**		
	Nausea	57
	Diarrhea	22
	Ineffectiveness	5
	Fatigue	3
	Renal dysfunction	3
	Bloating	2
	Headache	2
	Hypersensitivity	1
	Heartburn	1
	Hypoglycemia	1
	Mood changes	1
	Vomiting	1

### Drug and Adverse Drug Reaction Matching

The process was completed through consolidating the ADRs reported in the Twitter dataset to match the top 5 ADRs from SafeRx. Following the matching, a chi-square test was performed to test nonsignificant differences in the relative frequencies of an ADR between FAERS data and Twitter data. In order to demonstrate the similarity of Twitter’s ADR profile with that of FAERS, one should fail to reject H0 according to the *P* value from the chi-square test. Table 4 shows matched ADRs between the two data sources, relative frequencies of ADRs of each drug, and the results of chi-square test.

**Table 4:** Matched adverse drug reactions and chi-square test results for 5 drugs.

Drug and adverse drug events		Relative frequencies, FAERS^a^ data (%)	Relative frequencies, Twitter data (%)	Chi-square	*P* value
**Hydrocodone/acetaminophen**					
	Ineffectiveness	24.31	22.00	0.3	.60^b^
	Nausea	21.02	9.00	5.3	.02
	Fatigue	20.00	36.00	14.7	<.001
	Headache	15.13	2.00	13.2	<.001
**Prednisone**					
	Fatigue	23.41	5.00	18.8	<.001
	Dyspnea	18.76	1.00	47.0	<.001
**Amoxicillin**					
	Hypersensitivity	41.15	46.00	0.9	.35^b^
	Diarrhea	15.43	5.00	7.9	.005
	Nausea	15.18	8.00	3.8	.05^b^
	Fatigue	15.81	3.00	11.8	<.001
	Rash	12.42	16.00	1.0	.31^b^
**Gabapentin**					
	Ineffectiveness	28.55	22.00	2.1	.15^b^
	Fatigue	21.28	23.00	0.2	.68^b^
	Nausea	17.40	2.00	16.4	<.001
**Metformin**					
	Nausea	21.75	57.00	70.1	<.001
	Ineffectiveness	19.04	5.00	12.7	<.001
	Diarrhea	18.00	22.00	1.1	.30^b^
	Fatigue	15.56	3.00	11.9	<.001

^a^FAERS: US Food and Drug Administration Adverse Event Reporting System.

^b^Indicates a *P* value above .05, leading to the failure of rejecting the null hypothesis and indicating that there is no difference in ADR frequency reported between FAERS and Twitter.

## Discussion

### Principal Findings

Among the 5 drugs in the final analysis, a number of Twitter ADR relative frequencies were not significantly different from those of FAERS ADRs. For metformin, diarrhea was one of the side effects. As no significant difference was detected between FAERS and Twitter data on diarrhea (*P*=.30), it showed that Twitter ADR reports could be further studied for their use as a complementary ADR dataset. In the hydrocodone/acetaminophen group, there were no significant differences in ineffectiveness between sources (*P*=.60). Gabapentin was shown to comparatively result in ineffectiveness and fatigue according to FAERS and Twitter (*P*=.15 and *P=*.67, respectively). Three ADRs of amoxicillin, hypersensitivity, nausea, and rash, shared similar profiles on FAERS and Twitter (*P*=.35, *P=*.05, and *P=*.31, respectively).

ADRs remain one of the leading causes for preventable hospital admissions, reduced quality of life, increased financial burdens in the society, and mortality [19]. Prevention relies on adherence to evidence-based medicine, monitoring, medication therapy management, and pharmacogenomic testing [20]. Management of ADRs should emphasize effective prevention and timely detection, yet the current ADR reporting mechanism has shown delays in detection [21]. The cause for delays is multifactorial. Consumers might not know about such a reporting system, and the reporting steps could be troublesome. Further, as clinicians and patients are not required to report ADRs, many could be underreported. Social media and online resources have been proposed as additional resources for pharmacovigilance. In 2017, MacKinlay et al [22] evaluated ADRs of 3055 drugs on Twitter and found that Twitter had up to 72% precision of ADR detection. By extracting ADRs of erlotinib, nivolumab, and pembrolizumab through social health networks, Nikfarjam et al [23] detected that social media ADRs were comparable and 7 months ahead of ADRs from literature reports. Along with numerous major publications on validating ADR reports across different social media platforms, Hoang et al [24] took a step further and incorporated content authenticity and user credibility to improve ADR detection on Twitter. With more advanced technology for data mining and ADR detection, social media can serve as an additional channel for monitoring ADRs.

In this study, 10 drugs were identified, and ADR reports of these drugs on Twitter were retrospectively obtained by searching for tweets containing the drug names that mentioned ADR experiences. While adopting comparative methods used in previous studies, this study specifically focused on the 10 most commonly prescribed drugs to investigate if discrepancies existed pursuant to different drugs. Based on the results of this study, FAERS data and Twitter data showed some similar ADR profiles for hydrocodone/acetaminophen, amoxicillin, gabapentin, and metformin. In the data collection process, levothyroxine, alprazolam, lisinopril, and atorvastatin did not appear as keywords in sufficient tweets from March 1, 2016, to March 31, 2017. A possible explanation of the low number of tweets is the demographics of patients taking these medications. Atorvastatin, a lipid-lowering agent, is usually initiated for elderly patients, as are the antihypertensive agents lisinopril and amlodipine. Individuals aged 50 to 64 years and those older than 65 years represented 21% and 10% of all Twitter users, respectively [16]. Fewer Twitter users in these age ranges could potentially explain the low number of tweets for those drugs. The number of reports of these 3 drugs on FAERS further demonstrates that the lack of tweets was due to fewer users, as atorvastatin, lisinopril, and amlodipine had 6588, 5386, and 3192 reports on FAERS. Other social media–based studies have also experienced this challenge and achieved opposite conclusions due to inactivity for most of the drugs studied on social media [25,26]. Nevertheless, data from the remaining drugs indicates the potential role of Twitter as a complementary source of ADR reporting to FAERS.

The similarities observed for some ADRs between Twitter and FAERS data were disparate across the individual drugs studied. This variability further suggests that patients’ actual experiences with medications are not being shared with their providers or that providers have not reported these experiences to national ADR repositories at a similar rate. Moreover, the insufficiency of tweets for some drugs may indicate that social media ADR reporting should consider drug classes and the demographics of patients taking them. One recommendation is to further investigate social media ADR reporting for drugs that are consumed by a population that represents a large share of social media users and drugs that require early ADR detection.

In addition to being a supplementary data source for pharmacovigilance services, social media can also serve as a resource for pharmaceutical companies, regulatory bodies, researchers, health care professionals, patients, and policymakers. In this study, ineffectiveness appeared as an ADR for hydrocodone/acetaminophen, gabapentin, and metformin on both data sources. Gabapentin, for example, takes time to exert its full effect in controlling neurological pain. As 23.00% of Twitter ADRs and 28.55% of FAERS ADRs for gabapentin were ineffectiveness, it should encourage prescribers and pharmacists to consult patients on the time lag between taking the medication and seeing its effect. This study result should also prompt patient education on regular monitoring and diet adjustment when managing diabetes, as ineffectiveness for an antidiabetic drug, metformin, was 19.04% and 5.00% of all ADRs on FAERS and Twitter, respectively. Data mining to track ineffectiveness for hydrocodone/acetaminophen may offer a potential avenue for regulatory bodies in examining opioid use patterns.

### Limitations

This study does have two prominent limitations: sample size and search methodology. Among multiple social media platforms, only Twitter was selected as the data source. Despite Twitter’s users being from multiple age groups, patients may choose to share their ADR experiences on other sites such as Facebook, Instagram, Reddit, and online forums, which prevented this study from examining social media data across different platforms. Additionally, due to Twitter’s privacy setting, private tweets are not searchable, which can reduce the number of tweets for data collection. The sample size of tweets obtained for the drugs was relatively small compared with that of FAERS reports from March 1, 2016, to March 31, 2017. The sample size could be largely increased in future studies as Twitter contains a large collection of tweets. During the search process, the keywords hydrocodone/acetaminophen and Norco yielded more than 100 tweets in the time period, which could potentially improve the accuracy of Twitter ADR data. However, there was a lack of relevant tweets for 4 of the 10 drugs, even with the benchmark of 100 tweets. This situation could potentially be resolved by extending the time frame to more than 1 year; however, the extent of sample size improvement might not be significant given the low number of social media users when studying specific drugs such as atorvastatin and amlodipine.

Regarding the search mechanism, only one common brand name per drug was used to search for tweets, yet many drugs have multiple brand names. Lisinopril is sold under the brand names Prinivil and Zestril, and levothyroxine has brand names Synthroid, Levoxyl, and Thyrax. Using only one brand name in the study could limit the number of tweets obtained in this study, as patients might have shared their ADRs by using the brand names that were not included in this study. Other challenges to gathering all tweets through keywords include typographical errors, abbreviations, and unstructured lexicons. Furthermore, social media intrinsically bears a limitation in terms of patient follow-up. So far, research methodology involving social media pharmacovigilance has yet to be capable of investigating the causes of ADRs, the consequences of ADRs, and the actions taken to resolve ADRs. Some challenges are being tackled by computational technologies. For example, text normalization and classification through machine learning have been investigated by Sarker et al [27], and they offered insights into processing text data on social media. Other challenges of social media ADR reporting may continue to be barriers for taking full advantage of this data source.

Although social media cannot replace professional reporting systems such as FAERS at this stage, studies including this analysis have indicated the role of social media as a tool for early detection and a reporting system for mild symptoms. To demonstrate the accuracy and usability of social media ADR data in complementing FAERS, future studies may benefit by using a larger sample of data, including specific drugs, and assessing multiple social media platforms. It is also important to apply technology, along with structured reporting systems, to avoid arbitrary entries to better provide health care professionals, regulatory bodies, patients, and pharmaceutical companies with robust ADR data.

### Conclusion

While the use of Twitter as an ADR reporting platform has limitations, should be considered as a unique and complementary source of information rather than a validation tool of an existing ADR database. Future research should focus on validating Twitter and other social media platforms using involving larger sample sizes and different medications. Additionally, evaluating the types of ADRs on social media that share the most similarity with those on FAERS would be helpful to promote effective use of this source of information.

## Abbreviations

ADR: adverse drug reaction
CMSA: Center for Medication Safety Advancement
FAERS: FDA Adverse Event Reporting System
FDA: US Food and Drug Administration

## References

[1] Schatz S, Weber R, Murphy J (2015). Adverse drug reactions. Pharmacotherapy Self-assessment Program: CNS/Pharmacy Practice.

[2] Kohn L, Corrigan J, Donaldson M (2000). To err is human: building a safer health system.

[3] (2018). Preventable adverse drug reactions: a focus on drug interactions.

[4] Kongkaew C, Noyce PR, Ashcroft DM (2008). Hospital admissions associated with adverse drug reactions: a systematic review of prospective observational studies. Ann Pharmacother.

[5] Wu WK, Pantaleo N (2003). Evaluation of outpatient adverse drug reactions leading to hospitalization. Am J Health Syst Pharm.

[6] (2018). Questions and answers on FDA’s Adverse Event Reporting System.

[7] Golder S, Norman G, Loke YK (2015). Systematic review on the prevalence, frequency and comparative value of adverse events data in social media. Br J Clin Pharmacol.

[8] Schröder S, Zöllner YF, Schaefer M (2007). Drug related problems with Antiparkinsonian agents: consumer Internet reports versus published data. Pharmacoepidemiol Drug Saf.

[9] Duh MS, Cremieux P, Audenrode MV, Vekeman F, Karner P, Zhang H, Greenberg P (2016). Can social media data lead to earlier detection of drug-related adverse events?. Pharmacoepidemiol Drug Saf.

[10] Pages A, Bondon-Guitton E, Montastruc JL, Bagheri H (2014). Undesirable effects related to oral antineoplastic drugs: comparison between patients' internet narratives and a national pharmacovigilance database. Drug Saf.

[11] Smith K, Golder S, Sarker A, Loke Y, O'Connor K, Gonzalez-Hernandez G (2018). Methods to compare adverse events in Twitter to FAERS, drug information databases, and systematic reviews: proof of concept with adalimumab. Drug Saf.

[12] Gliklich R, Dreyer N, Leavy M, Agency for Healthcare Research and Quality (2014). Adverse event detection, processing, and reporting. Registries for Evaluating Patient Outcomes: A User's Guide. 3rd Edition.

[13] (2017). The GoodRx Top 10.

[14] Freifeld CC, Brownstein JS, Menone CM, Bao W, Filice R, Kass-Hout T, Dasgupta N (2014). Digital drug safety surveillance: monitoring pharmaceutical products in twitter. Drug Saf.

[15] Chan B, Lopez A, Sarkar U (2015). The canary in the coal mine tweets: social media reveals public perceptions of non-medical use of opioids. PLoS One.

[16] Manchikanti L, Helm S, Fellows B, Janata JW, Pampati V, Grider JS, Boswell MV (2012). Opioid epidemic in the United States. Pain Physician.

[17] Greenwood S, Perrin A, Duggan M (2016). Social Media Update 2016.

[18] Twitter Advanced Search.

[19] Formica D, Sultana J, Cutroneo PM, Lucchesi S, Angelica R, Crisafulli S, Ingrasciotta Y, Salvo F, Spina E, Trifirò G (2018). The economic burden of preventable adverse drug reactions: a systematic review of observational studies. Expert Opin Drug Saf.

[20] Cacabelos R, Cacabelos N, Carril JC (2019). The role of pharmacogenomics in adverse drug reactions. Expert Rev Clin Pharmacol.

[21] Ma P, Marinovic I, Karaca-Mandic P (2015). Drug manufacturers' delayed disclosure of serious and unexpected adverse events to the US Food and Drug Administration. JAMA Intern Med.

[22] MacKinlay A, Aamer H, Yepes AJ (2017). Detection of adverse drug reactions using medical named entities on Twitter. AMIA Annu Symp Proc.

[23] Nikfarjam A, Ransohoff JD, Callahan A, Jones E, Loew B, Kwong BY, Sarin KY, Shah NH (2019). Early detection of adverse drug reactions in social health networks: a natural language processing pipeline for signal detection. JMIR Public Health Surveill.

[24] Hoang T, Liu J, Pratt N, Zheng VW, Chang KC, Roughead E, Li J (2018). Authenticity and credibility aware detection of adverse drug events from social media. Int J Med Inform.

[25] Caster O, Dietrich J, Kürzinger M, Lerch M, Maskell S, Norén GN, Tcherny-Lessenot S, Vroman B, Wisniewski A, van Stekelenborg J (2018). Assessment of the utility of social media for broad-ranging statistical signal detection in pharmacovigilance: results from the WEB-RADR Project. Drug Saf.

[26] van Stekelenborg J, Ellenius J, Maskell S, Bergvall T, Caster O, Dasgupta N, Dietrich J, Gama S, Lewis D, Newbould V, Brosch S, Pierce CE, Powell G, Ptaszyńska-Neophytou A, Wiśniewski AFZ, Tregunno P, Norén GN, Pirmohamed M (2019). Recommendations for the use of social media in pharmacovigilance: lessons from IMI WEB-RADR. Drug Saf.

[27] Sarker A, Belousov M, Friedrichs J, Hakala K, Kiritchenko S, Mehryary F, Han S, Tran T, Rios A, Kavuluru R, de Bruijn B, Ginter F, Mahata D, Mohammad SM, Nenadic G, Gonzalez-Hernandez G (2018). Data and systems for medication-related text classification and concept normalization from Twitter: insights from the Social Media Mining for Health (SMM4H)-2017 shared task. J Am Med Inform Assoc.

